# Molecular characterization of the *tet* (M)-carrying transposon Tn*7124* and plasmids in *Escherichia coli* isolates recovered from swine

**DOI:** 10.3389/fvets.2024.1430398

**Published:** 2024-10-23

**Authors:** Yingying Liu, Zhu Qiao, Yan Ma, Mingcheng Wang, Gongzheng Hu, Enzhong Li

**Affiliations:** ^1^School of Biological and Food Processing Engineering, Huanghuai University, Zhumadian, China; ^2^College of Animal Husbandry and Veterinary Science, Henan Agricultural University, Zhengzhou, China

**Keywords:** IncR-type plasmid, *tet* (M), insertion sequence 26, transposon 7124, *Escherichia coli*

## Abstract

Here, we report the genetic features and evolutionary mechanisms of two *tet* (M)-bearing plasmids (pTA2 and pTA7) recovered from swine *Escherichia coli* isolates. The genetic profiles of pTA2 and pTA7 and corresponding transconjugants were accessed by S1 nuclease pulsed-field gel electrophoresis and Southern hybridization, followed by whole genome sequencing and bioinformatics analysis. The biological influences of pTA2 and pTA7 were determined by stability and direct competition assays. Both pTA7 and pTA2 had the IncR backbone sequences but differed in the multidrug resistance region (MDR). The MDR of pTA2 consisted of *sul3*, *tet* (M), *qnr*S1, *bleO*, *oqxAB*, *floR*, *aadA1*, *cmlA1*, *aadA2*, and *tet* (A)-*tetR* (A) in addition to 22 insertion sequences. Notably, pTA2 carried the novel complex Tn*7124* (IS*26*-ctp-lp-*tet* (M)-hp-IS*406*tnp-IntI4-IS*26*) harboring *tet* (M). The fragment carrying *tet* (M) (IS*26-ctp-*lp-*tet* (M)-IS*406 tnp-ctp-aadA1*-*cmlA1*-*aadA2*-*dfrA12-*Int*I1*), named Tn*6942-like*, and the two resistance modules IS*Vsa3*-*VirD2*-*floR*-*lysR* and *tet* (A)-*tetR* (A) were located in the MDR of pTA7. Both pTA2 and pTA7 were highly stable in *E. coli* DH5α cells with no fitness cost to the host or disadvantage in growth competition. These results indicate that transposons carrying *tet* (M) continuously integrate via mediation with an insertion sequence, which accelerates the transmission of *tet* (M) in *E. coli* isolates through integration of other drug-resistant genes, thereby posing a potential serious threat to the efficacy of clinical treatment.

## 1 Introduction

The tetracycline resistance gene *tet* (M), which is widely distributed among Gram-positive bacteria, imparts resistance to tetracyclines via a ribosomal protection mechanism. To date, *tet* (M) has been identified in 38 genera of Gram-positive bacteria and 39 genera of Gram-negative bacteria (1–3). In most bacteria, *tet* (M) is associated with other tetracycline resistance genes, likely involving conjugative transposons located on the chromosome and conjugative plasmids (4–6). In Gram-positive bacteria, *tet* (M) has been linked to 23 transposons, including Tn*916*/Tn*1545*-like in *Enterococcus* spp. (7), Tn*5801*-like in *Staphylococcus aureus* and *Enterococcus faecalis* (6, 8), and Tn*5397*-like in *Clostridium difficile* and *Enterococcus faecium* (9, 10). Among these, Tn*916*, which was the first conjugative transposon found to covey resistance to antibiotics (1), harbors 24 open reading frames that are organized into functional modules involved in conjugation transfer, recombination (excision and insertion responses), transcriptional regulation, and auxiliary functions (antibiotic resistance) (11, 12). Subsequently, Tn*1545*, which was identified in *Streptococcus pneumoniae,* is highly homologous to Tn*916* (difference of only one nucleotide), especially between the ~2,000-bp integrase and cleavase genes that encode translocation functions. Also, Tn*1545* harbors three resistant genes [*tet* (M), *ermAM,* and *aphA-3*] (13, 14), while Tn*916* only harbors *tet* (M). The similarity in the *tet* (M) sequence between Tn*1545* and Tn*916* is reportedly 94.5% (15), demonstrating that *tet* (M) is evolutionarily conserved across different species of bacteria. Tn*5801*, a member of the Tn*916* family, was initially isolated from *Staphylococcus aureus* in 2001 (16). Afterward, different types of Tn*5801*-like gene islands have been identified in *Enterococcus*, *Lactobacillus*, *Lactococcus*, *Staphylococcus*, *Streptococcu*s, and *Clostridium* isolates from humans, animals, and food products in Europe, the United States, Asia, and Australia, suggesting that the element carrying *tet* (M) has spread among Gram-positive species worldwide (6, 17–21). Transmission of *tet* (M) is mediated by transposons and plasmids. However, the gene environment of *tet* (M) remains unclear.

Although various transposons carrying *tet* (M) have been extensively reported in Gram-positive bacteria, the mode of transmission in Gram-negative bacteria remains unclear. Our group previously reported that *tet* (M) was linked to Tn*6539* in *E. coli*, Tn*6709* and Tn*6942* in *Salmonella* (22–24). Here, we report the novel *tet* (M)-bearing Tn*7124* and a Tn*6942-like* fragment carried by pTA2 and pTA7, respectively, and clarify the molecular, genetic, and biological characteristics of pTA2 and pTA7 in *E. coli* DH5α cells.

## 2 Materials and methods

### 2.1 Bacterial strains

Two *tet* (M)-positive *E. coli* strains (A2 and A7) isolated from the feces of pigs in Henan province, China, in December 2017 were identified using the VITEK 2 automated bacterial identification and susceptibility testing system (bioMérieux, Marcy-l’Étoile, France). *E. coli* strains J53 (sodium azide resistant), DH5α, and ATCC 25922 were stored in our laboratory.

### 2.2 Transformation experiments

Competent *E. coli* DH5α cells were transformed with the plasmids of the donor strains A2 and A7 by electroporation. The transformants were screened on Luria–Bertani (LB) agar plates containing doxycycline (16 mg/L). The conjugation frequency was calculated as the ratio of the number of transconjugants per recipient. Both transformants were confirmed by S1 nuclease pulsed-field gel electrophoresis (S1-PFGE). The presence of *tet* (M) in the transformants was confirmed by polymerase chain reaction analysis and whole genome sequencing.

### 2.3 Antimicrobial susceptibility testing

The susceptibility of three *tet* (M)-positive isolates and corresponding transformants to 13 antibiotics (i.e., amoxicillin, ceftiofur, cefquinome, gentamicin, amikacin, oxytetracycline, doxycycline, florfenicol, colistin, trimethoprim-sulfoxazole, enrofloxacin, olaquindox, and mequindox) was determined by the broth micro dilution method in accordance with the guidelines of the Clinical and Laboratory Standards Institute (25, 26). *E. coli* ATCC 25922 was used for quality control.

### 2.4 S1-PFGE and southern hybridization

DNA from the donor strains and transformants were treated with S1 nuclease and then separated by PFGE. The location of *tet* (M) was determined by Southern hybridization.

### 2.5 Plasmid sequencing and annotation

Plasmids pTA2 and pTA7 were extracted from the transformants ZA2 and ZA7 using the QIAGEN^®^ Plasmid Midi Kit (Qiagen GmbH, Hilden, Germany). Whole genome sequencing of A2 and A7 were conducted a HiSeq™ Sequencing System (Illumina, Inc., San Diego, CA, United States) and Oxford Nanopore Technologies (ONT) MinION platforms (Oxford Nanopore Technologies Ltd., Oxford) with 400 bp paired-end reads. The obtained sequences were assembled using unicycler 0.5.0 and Flye 2.9.1^1^ software with the hybrid strategy. The pTA2 and pTA7 sequences were initially predicted and annotated using the RAST (Rapid Annotation using Subsystem Technology) server (v2.0),^2^ and corrected manually using the Basic Local Alignment Search Tool (BLAST) algorithm.^3^ The plasmids replicon genotype were identified using PlasmidFinder 2.1.^4^ Insertion sequence elements were recovered using ISfinder.^5^ Contigs containing resistance genes were ascertained using the comprehensive antibiotic resistance database (CARD).^6^ Comparative analysis was conducted and plasmid maps were generated using the Easyfig application^7^ and the BLAST Ring Image Generator.^8^

### 2.6 Biological characteristics of pTA2 and pTA7

The stability of pTA2 and pTA7 in *E. coli* DH5α cells cultured in antibiotic-free LB broth were determined as previously described (27). Briefly, a 100-μL aliquot of suspended bacterial cells was diluted in LB broth and plated on LB agar. Then, 20 colonies were randomly chosen and replica plated onto LB agar plates with 16 μg/mL doxycycline, and the presence of the *tet*(M) gene were confirmed by PCR.

The bacterial growth kinetics of *E. coli* ZA2 (ZA7) and DH5α cells were observed by culturing overnight in LB medium with and without doxycycline. Then, 10^7^ colony-forming units (CFUs) were added independently into 20 mL of fresh LB medium with and without doxycycline and cultured at 37°C for 12 h. Absorbance at 600 nm was measured every hour.

The fitness costs of pTA2 and pTA7 were determined by competition assays of *E. coli* ZA2 (ZA7) and DH5α cells as previously described. Briefly, *E. coli* ZA2 (ZA7) and DH5α cells were cultured in 20 mL of LB broth at 37°C for 16 h. Then, 2 × 10^6^ CFUs of *E*. *coli* ZA2 (ZA7) and DH5α cells were cultured in 20 mL of antibiotic-free LB broth at 37°C with continuous shaking (120 rpm). Finally, 4 × 10^6^ CFUs were transferred to 20 mL of fresh LB broth every 24 h. Samples were collected every hour during the first 12 h and then every 24 h for 7 days. The CFUs of each sample on LB agar plates with and without doxycycline were quantified.

### 2.7 Nucleotide sequence accession number

The complete sequences of pTA2 and pTA7 in addition to chromosome A2 and A7 were submitted to the GenBank database^9^ under accession numbers CP069710, CP069708, CP069711, and CP069709, respectively.

## 3 Results and discussion

### 3.1 Characterization of *Escherichia coli* strains A2 and A7

Although transformation of *E. coli* strains A2 and A7 by conjugation was not possible, *E. coli* DH5α cells were successfully transformed by electroporation at the same frequency of 1 × 10^−6^. Strains A2 and A7, and the corresponding transconjugants ZA2 and ZA7, were resistant to tetracycline, oxytetracycline, florfenicol, and amoxicillin, while A2 and ZA2 were resistant to sulfamethoxazole-trimethoprim (Table 1). S1-PFGE showed that a single plasmid was obtained from the transconjugants ZA2 and ZA7 (designated as pTA2 and pTA7, respectively). As shown in Supplementary Figures S1, S2, Southern hybridization indicated that *tet*(M) was located on pTA2 (~110 kb) and pTA7 (~70 kb).

**Table 1:** The MICs of A2, A7 and their transconjugants ZA2, ZA7.

Isolates	AMC	FFC	AN	COL	CEF	CEQ	GM	ST	EN	DOX	OXY	TET
A2	>512	>512	1	<0.5	<0.5	<0.5	1	>512	0.5	32	128	64
ZA2	>512	256	1	<0.5	<0.5	<0.5	1	>512	0.5	16	128	64
A7	>512	256	<0.5	<0.5	<0.5	<0.5	2	2	<0.5	32	128	256
ZA7	>512	128	<0.5	<0.5	<0.5	<0.5	1	2	<0.5	16	16	4
DH5α	1	<0.5	<0.5	<0.5	1	1	1	0.5	1	<0.5	1	<0.5

### 3.2 Sequence analysis of plasmids in *Escherichia coli* strains A2 and A7

**Figure 1:**
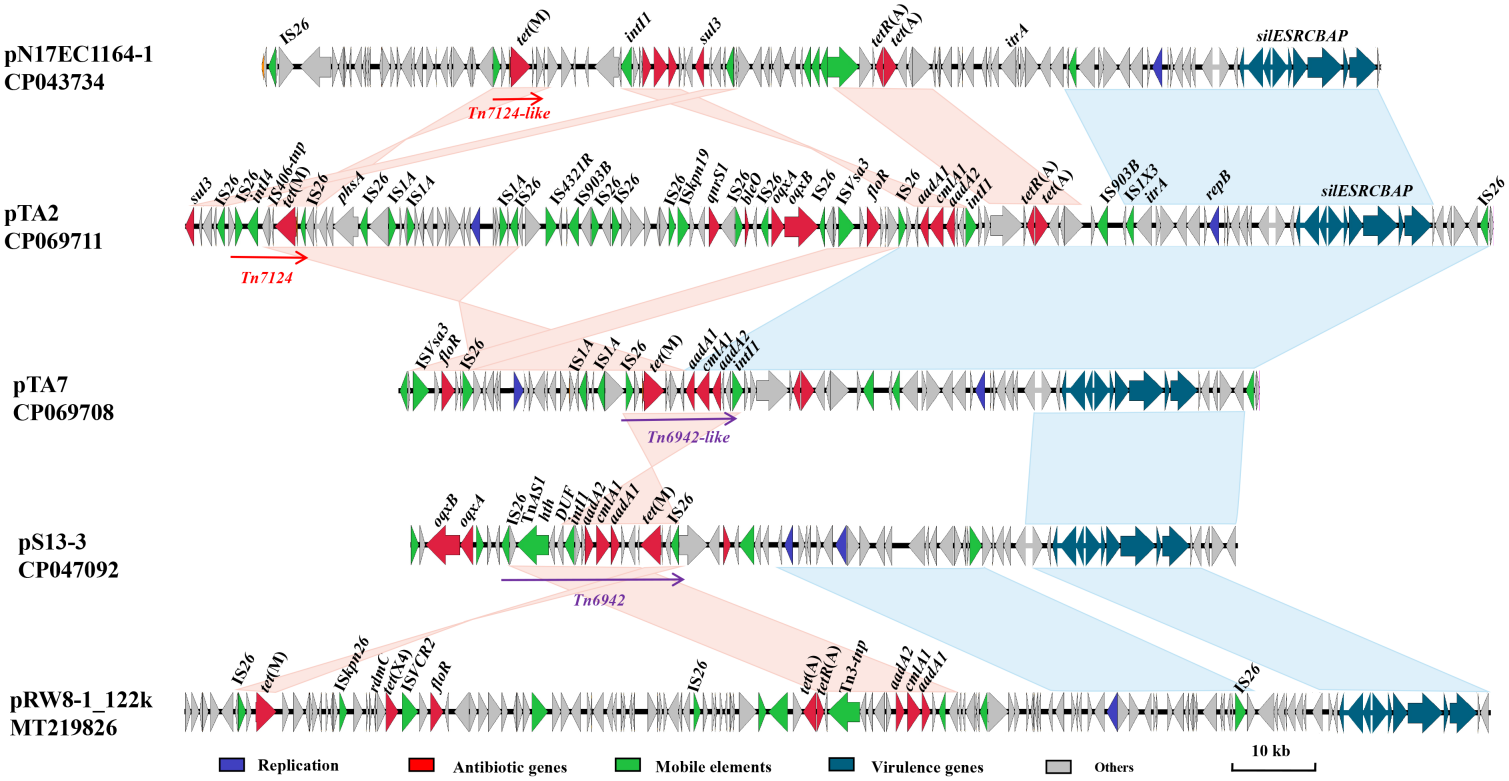
Comparative analysis of pTA2, pTA7 with other plasmids including pN17EC1163–1, pS13-3, pRW8_122k. Replicon genes are in blue; resistance genes in red; mobile elements in green; the loci of virulence-associated genes (*sil*ESRCBAP) in teal; and hypothetical proteins in gray.

Whole genome sequencing showed that the chromosome of strain A2 comprised 4,761,946 bp (4,593 protein-coding regions) and harbored one copy of pTA2 (120,379 bp), while the chromosome of strain A7 comprised 4,620,143 bp (4,423 protein-coding regions) and harbored one copy of pTA7 (77,243 bp). The genome of A2 had more than 30 mobile elements, which included IS*1A*, IS*Ec17*, IS*609*, IS*150*, and IS*1H*, while the genome of A7 had more than 90 mobile elements, which mainly included IS*Kpn26* and IS*1R* (Figure 1).

**Figure 2:**
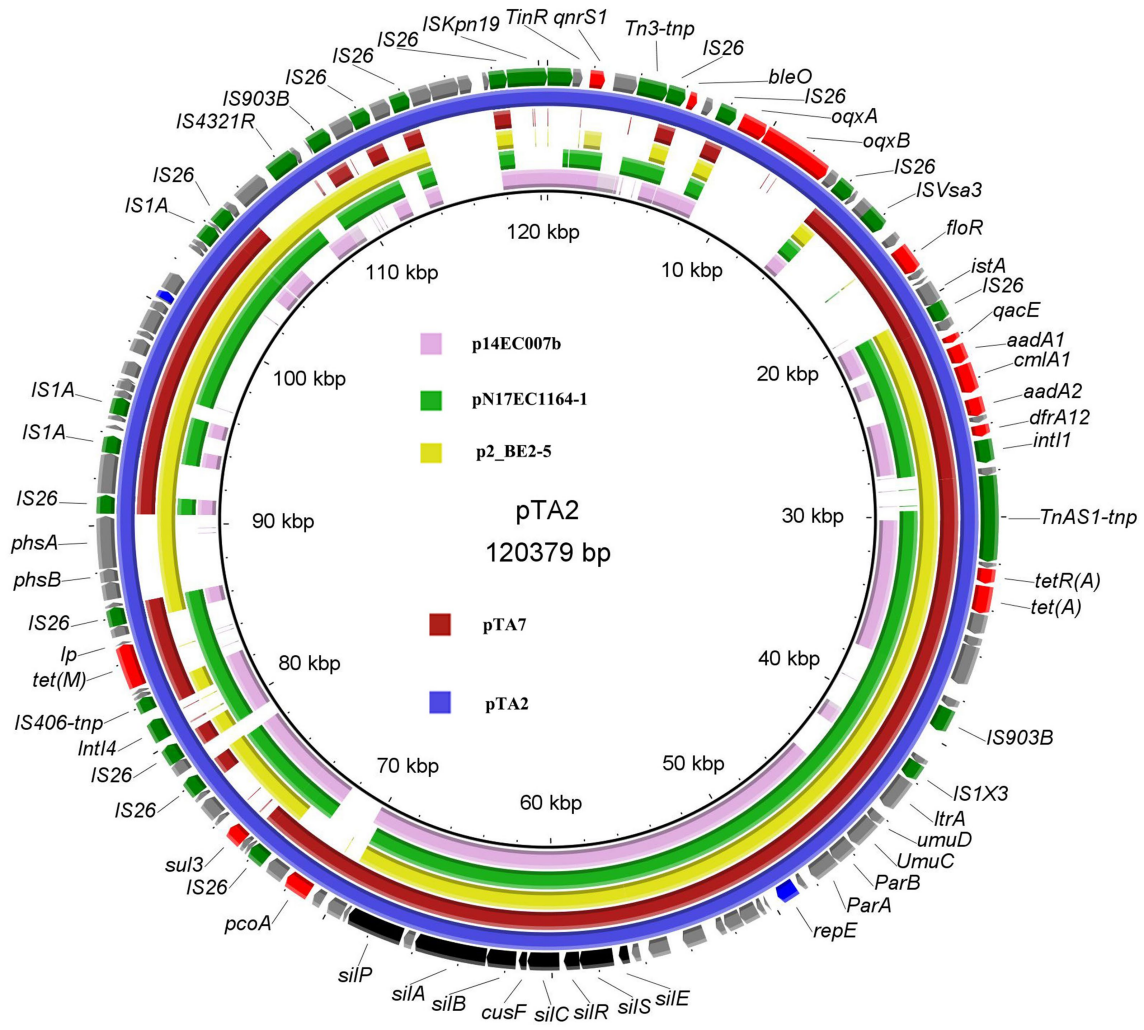
Structural comparisons of pTA2, pTA7 and the similar plasmids. The outer ring represents the CDSs of the reference sequence pTA2. The similar plasmids including the *E.coli* plasmids pN17EC1163-1 (IncR-X1, CP043734), p14EC007b (CP024133) and p2_BE2-5 (CP032988).

BLASTn analysis indicated that pTA2 was 99.75% sequence similarity with pTA7 at 72% coverage, 99.95% at 73% coverage with *E. coli* p2_BE2-5 (CP032988) isolated from an egret in Chengdu, China, 99.76% at 72% coverage with *E. coli* pN17EC1163–1 (IncR-X1, CP043734) isolated from swine in the United States, and 99.20% at 56% coverage with *E. coli* p14EC007b (CP024133) isolated from clinical patients in Guangdong, China (Figure 2). In addition to the three plasmids mentioned above, only 12 strains exhibited higher sequence similarity with pTA2 (99.2% at 51–66% coverage), which included 11 *E. coli* strains and 1 *Salmonella* isolate from swine, and there were only three plasmids isolated from *E. coli* strains obtained from animals, including plasmid pYUXJMC1-2, (CP125352, chicken meat), plasmid unnamed, (CP038858, feces of pig) and plasmid unnamed11 (CP122915, feces of *Sus scrofa*). Compared to plasmids pTA2 and pTA7, they just shared backbone sequences of IncR plasmid. While pTA2 shared lower sequence similarity with other plasmids in the NCBI database. Although reports of pTA2 and pTA7-like plasmids are limited, the majority of 17 plasmids isolated in China exhibited regional characteristics among various hosts, indicating wide circulation.

Comparative sequence analysis demonstrated that pTA2 and pTA7 were the IncR type, with GC contents of 51%, and the same replication initiator *repB*, which shared 100% amino acid identity to the corresponding region of pK245 of *Klebsiella pneumoniae* strain DQ449578 isolated from a patient in Taiwan, China, in 2002. Both pTA2 and pTA7 had typical IncR plasmid backbone genes, including *repB*, *parAB*, and *umuCD*, but not *resD*, and the downstream virulence gene *silESRCBAP* (Figure 1).

The most notable difference between pTA2 and pTA7 was the MDR region. The MDR region of pTA7 consisted of three resistance modules, IS*Vsa3*-*VirD2*-*floR*-*lysR*, insertion sequence (IS)*26-ctp-*lp-*tet*(M)-IS*406tnp-ctp-aadA1*-*cmlA1*-*aadA2*-*dfrA12-*Int*I1*, and *tet*(A)-*tetR*(A), and several insertion sequences. The fragment carrying *tet*(M) was 2,300 kb in length. The *tet*(M)-bearing structure Tn*6942* was similar to pS13-3 (CP047092) of *Salmonella enterica*, but lacked the helix-inverse-helix domain, Tn*3* family transposase, and IS*26*, which were located downstream of *insI1* in pTA7. Therefore, the fragment was named Tn*6942*-like. The MDR region of pTA2 consists of 11 resistance genes, including *sul3*, *tet*(M), *qnrS1*, *bleO*, *oqxAB*, *floR*, *aadA1*, *cmlA1*, *aadA2*, and *tet*(A)-*tetR*(A), and 22 insertion sequences, which included IS*26,* IS*1A,* IS*903B*, IS*Vsa3*, IS*Kpn19,* IS*4321R*, and IS*1X*. Notably, pTA2 contained *sul3*, *qnr*S1, *bleO*, and an *oqxAB*-bearing resistance module along with several insertion sequences upstream of IS*Vsa3*, while the other sequences were almost consistent with pTA7 (Figure 1).

In addition, the fragment containing *tet*(M) slightly differed between pTA7 and pTA2. The *tet*(M) sequence was located at the composite Tn*7124* between two IS*26*-bracked composites, as determined in reference to the Transposon Nomenclature Database.^10^ Tn*7124* is 7.2 kb in length and organized as IS*26*, conjugative transfer protein ctp, *tet*(M) leader peptide gene, *tet*(M), hypothetical protein (hp), integrase (*Int*)*I4*, ctp, and IS*26* (Figure 1). Comparative sequence analysis showed that fragment IS*26-ctp-*lp-*tet*(M) was relatively conserved and shared 100% sequence identity with the sequences of pN17EC1163–1 and pRW8-1_122k (Figure 1), while the downstream sequence of *tet*(M) was hp-IS*406* tnp-IntI3*-*hp, which is same sequence as Tn*7124-like* of pN17EC1163–1.

Although usually coded by the bacterial chromosome, *tet*(M) was also located on the conjugative plasmids IncHI2, IncFII, IncFI, IncN1-IncHI2, and IncX1-FI isolated from *Campylobacter jejuni*, *Neisseria meningitidis*, *Kingella denitrificans*, *Aikenella erosus*, *Clostridium percapsulatus*, *Salmonella,* and *E. coli* (28, 29). Hence, conjugation plasmids can facilitate horizontal transfer of *tet*(M) genes between species. IncR plasmids were initially identified as a novel group of incompatible plasmids in 2009 (30). Subsequent studies have reported that conjugation plasmids are broadly distributed in clinically relevant strains of *Enterobacteriaceae* and *Klebsiella pneumoniae*, which intensified the fitness of the host cell by conferring resistance to fluoroquinolones (*qnrS1* and *qnrB4*), aminoglycosides (*armA*), and beta-lactams (*bla*_KPC-2_, *bla*_DHA-1_, *bla*_NDM-1_, and *bla*_VIM-1_) and carbapenems (31–34). In addition, IncR plasmids often coexist with other replicants, such as IncC, IncN, IncHI, and IncFII. Thus, the resistance reservoir carried by IncR plasmids may spread to transferable plasmids through translocation or plasmid recombination events, thereby contributing to the high plasticity of multiple replicant plasmids (35). The complex class 1 integrons and *IS*s located on pTA2 and pTA7 are formidable gene-capturing tools that can mobilize extremely large sections of DNA encoding a variety of antibiotic resistance genes (36). Therefore, *tet*(M)-bearing pTA2 and pTA7 may have spread between different species of bacteria and conferred resistance to tetracycline.

In this study, the *tet*(M)-bearing complex Tn*7124* had two IS*26* elements in opposite directions at boundaries. IS*26*, a member of the IS*6* insertion sequence family, transmits antibiotic resistance genes in Gram-negative bacteria primarily through the formation of complex transposons, of which most contain a central region containing the resistance gene and two IS*26* elements at the boundaries in the same or opposite directions (37). In Gram-negative bacteria, IS26 elements are usually found at the boundary of the transposon, but there is no marker of IS26 integration on either side of the 8-bp target point repeat (38, 39). Therefore, complex transposons are not formed by metastasis, but rather homologous recombination. In 2014, Harmer et al. proposed a new resistance gene mechanism model mediated by IS*26*, which considered IS*26* and resistance genes as mobile units (TU), a new family of mobile genetic (40). The TU released from the gene sequence can recognize another adjacent IS*26* as a target site in the same direction, which resulted in tandem arrangement of IS*26* elements and the formation of complex transposons.

The frequency of TU-mediated co-integration formation was 60 times higher than that of single IS*26*-mediated co-integration formation, indicating that intact IS*26* elements in the gene sequence can recruit IS*26*-mediated resistance genes. TU integration can occur through reactions catalyzed by Tnp (transposase of IS*26*) or by *recA*-dependent homologous recombination. However, the reaction catalyzed by tnp is 100 times more efficient than *recA*-dependent homologous recombination. In addition, analysis of different patterns of the flanking sequences of 70 IS*26* copies in 8 genomes of carbapenemase-producing *Enterobacteriaceae* found that IS*26* promoted rearrangement of drug-resistant plasmids through inter-and intramolecular replication translocations (41). Despite repeated attempts, the closed circular intermediate IS*26-*ctp-lp-*tet*(M)-hp-IS*406* tnp-IntI3*-*hp of Tn*7124* was not detected in the A2 strain and no direct repeats were found flanking the IS*26* element in Tn*7124*, suggesting that Tn*7124* may have occurred by recombination, rather than transposition, which was likely controlled by the IS*26* element.

### 3.3 Biological characteristics of pTA2 and pTA7

**Figure 3:**
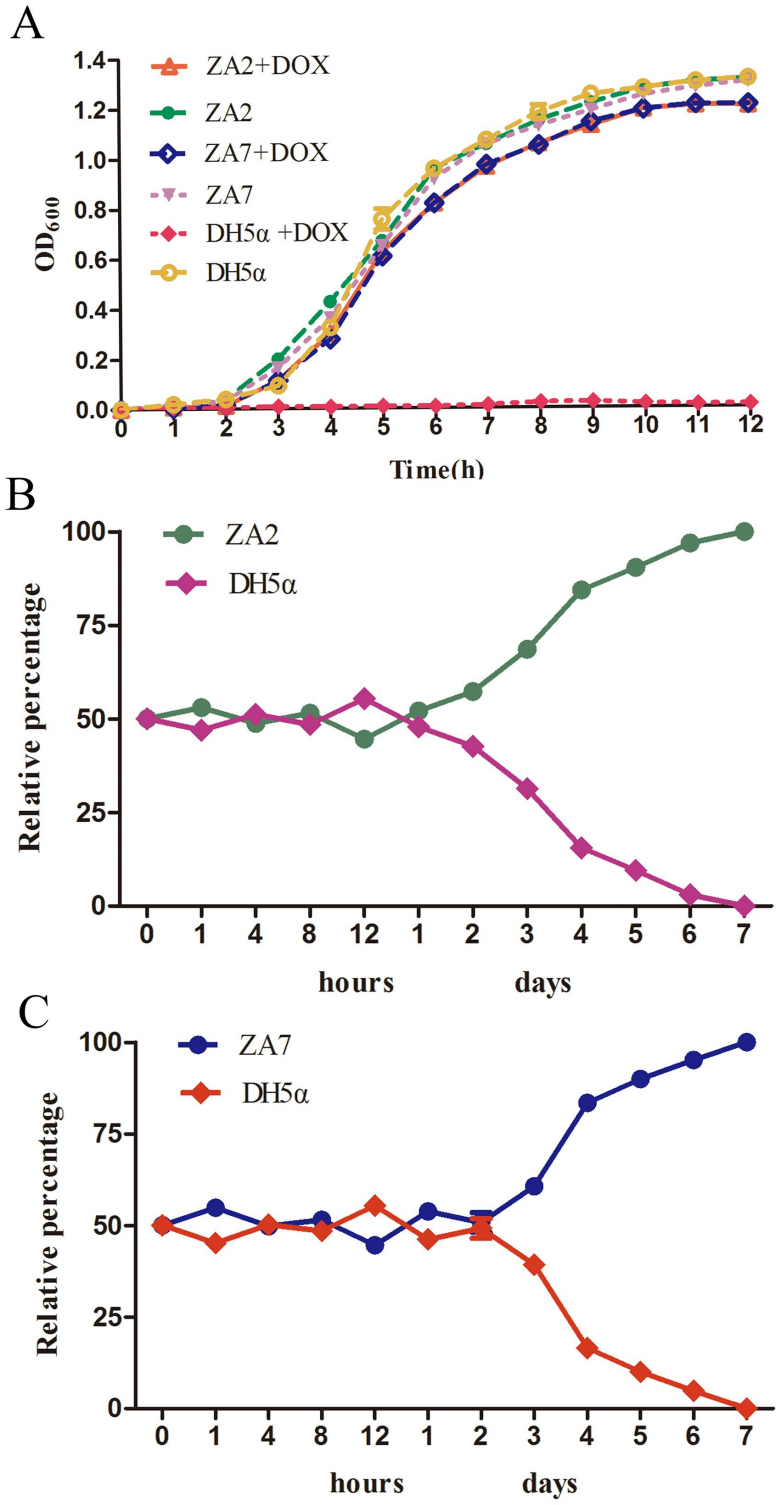
(A) The growth kinetics of strains ZA2, ZA7and DH5α in LB broth in the presence and absence of 16 ug/ml doxycycline. The standard deviations of the mean of three independent experiments represented by error bars. (B) The competition profiles of strains ZA2 and DH5α, the initial ratio was equal. The relative percentage of each strain at different time points is shown. The competition curves were constructed with the mean of three independent experiments that the standard deviation represented by error bars. (C) The competition profiles of strains ZA7 and DH5α, the initial ratio was equal. The relative percentage of each strain at different time points is shown. The competition curves were constructed with the mean of three independent experiments that the standard deviation represented by error bars.

pTA2 and pTA7 were highly stable (100%) for 14 days in *E. coli* DH5α cells cultured in antibiotic-free medium, indicating that both carried the resistance genes *tet*(M), *tet*(A), *floR*, *aadA1*, *cmlA1*, *aadA2*, and *sul2*. As shown in Figure 3A, ZA2 and ZA7 exhibited similar growth as *E. coli* strain DH5α in LB broth without doxycycline, while the growth rates of ZA2 and ZA7 were slightly increased in LB broth without doxycycline than with doxycycline. *E. coli* strain DH5α failed to grow in the presence of doxycycline. The growth kinetics obtained over 12 h showed no evidence of any fitness cost exacted by pTA2 and pTA7.

Further studies on the fitness cost of plasmids carrying *tet*(M) will be of great interest to determine the importance of these plasmids in tetracycline resistance of *E. coli*. As shown in Figures 3B,C, equal numbers of bacteria with and without plasmids were mixed and subcultured every 24 h for 7 days, and the proportion of bacteria carrying plasmids was monitored every hour for the first 12 h. During this period, the ratio was almost 1:1 at every time point, whereas ZA2 outnumbered *E. coli* DH5α after co-culture for 2 days and ZA7 after 3 days. Afterward, the number of *E. coli* DH5α cells in the mixed culture continuously decreased. At 7 days, the proportions of ZA2 and ZA7 decreased to less than 1%. The transconjugants ZA2 and ZA7 showed competitive advantages relative to *E. coli* DH5α throughout the entire experiment.

## 4 Conclusion

The novel *tet*(M)-bearing resistance modules Tn*7124* and Tn*6942-like* were found in pTA2 and pTA7 isolated from *E. coli* strains A2 and A7. Transfer of *tet*(M) occurred with the resistant genes *sul3*, *qnr*S1, *bleO*, *oqxAB*, *floR*, *aadA1*, *cmlA1*, *aadA2*, and *tet*(A)-*tetR*(A) in the novel self-transmissible pTA2, and with *floR*, *aadA1*, *cmlA1*, *aadA2*, and *tet*(A)-*tetR*(A) in pTA7, suggesting that pTA2 and pTA7 may act as reservoirs for these genes. Hence, these IncR plasmids likely facilitate the dissemination of *tet*(M) and other resistance genes. Therefore, there is an urgent need to strengthen surveillance and efforts to limit the spread of this MDR plasmid among *Enterobacteriaceae*, as well as to enhance surveillance of antibiotic resistance outwards the hospital settings, because studying resistance into the hospital setting had no result on fighting resistance, all these years. Typing of clones, plasmids and transposons derived from food producing animals could reveal MDR origins and their circulation between humans and animals.

## Data Availability

The datasets presented in this study can be found in online repositories. The names of the repository/repositories and accession number(s) can be found in the article/Supplementary material.

## Footnotes

^1^https://github.com/fenderglass/Flye

^2^https://rast.nmpdr.org/

^3^http://blast.ncbi.nlm.nih.gov/blast

^4^https://cge.food.dtu.dk/services/PlasmidFinder/

^5^https://isfinder.biotoul.fr/

^6^https://card.mcmaster.ca

^7^https://mjsull.github.io/Easyfig/

^8^https://sourceforge.net/projects/brig/

^9^https://www.ncbi.nlm.nih.gov/genbank/

^10^https://transposon.lstmed.ac.uk

## Supplementary material

Supplementary Figure S1 (a) S1-PFGE of *E. coli* A2 and its transconjugant ZA2. (b) Southern hybridization of *E. coli* A2 and ZA2 with the *tet*(M) gene as the probe. Marker, *Salmonella* Braenderup H9812.

Supplementary Figure S2 (a) S1-PFGE of *E. coli* A7 and its transconjugant ZA7. (b) Southern hybridization of *E. coli* A7 and ZA7 with the *tet*(M) gene as the probe. Marker, *Salmonella* Braenderup H9812.
