# Supplementary figures and images for: Molecular characterization of the tet (M)-carrying transposon Tn7124 and plasmids in Escherichia coli isolates recovered from swine
Source: Front Vet Sci. 2024 Oct 23;11:1430398. doi: 10.3389/fvets.2024.1430398 (PMC11539080; doi:10.3389/fvets.2024.1430398)

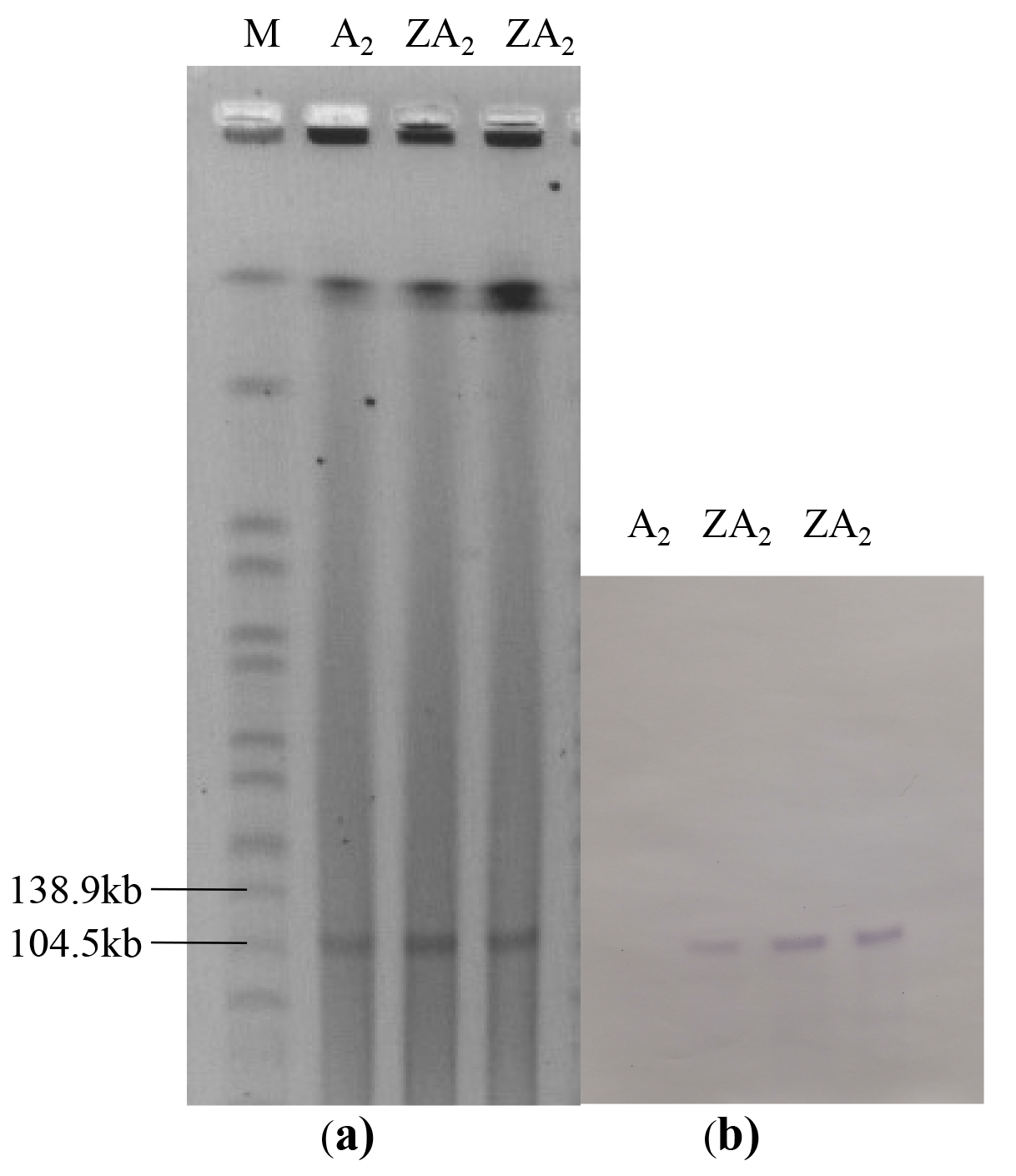

Supplement: Supplementary Figure S1 — (a) S1-PFGE of E. coli A2 and its transconjugant ZA2. (b) Southern hybridization of E. coli A2 and ZA2 with the tet(M) gene as the probe. Marker, Salmonella Braenderup H9812. [file Data_Sheet_1.ZIP › Supplementary files/FIGURE.S1.tif]

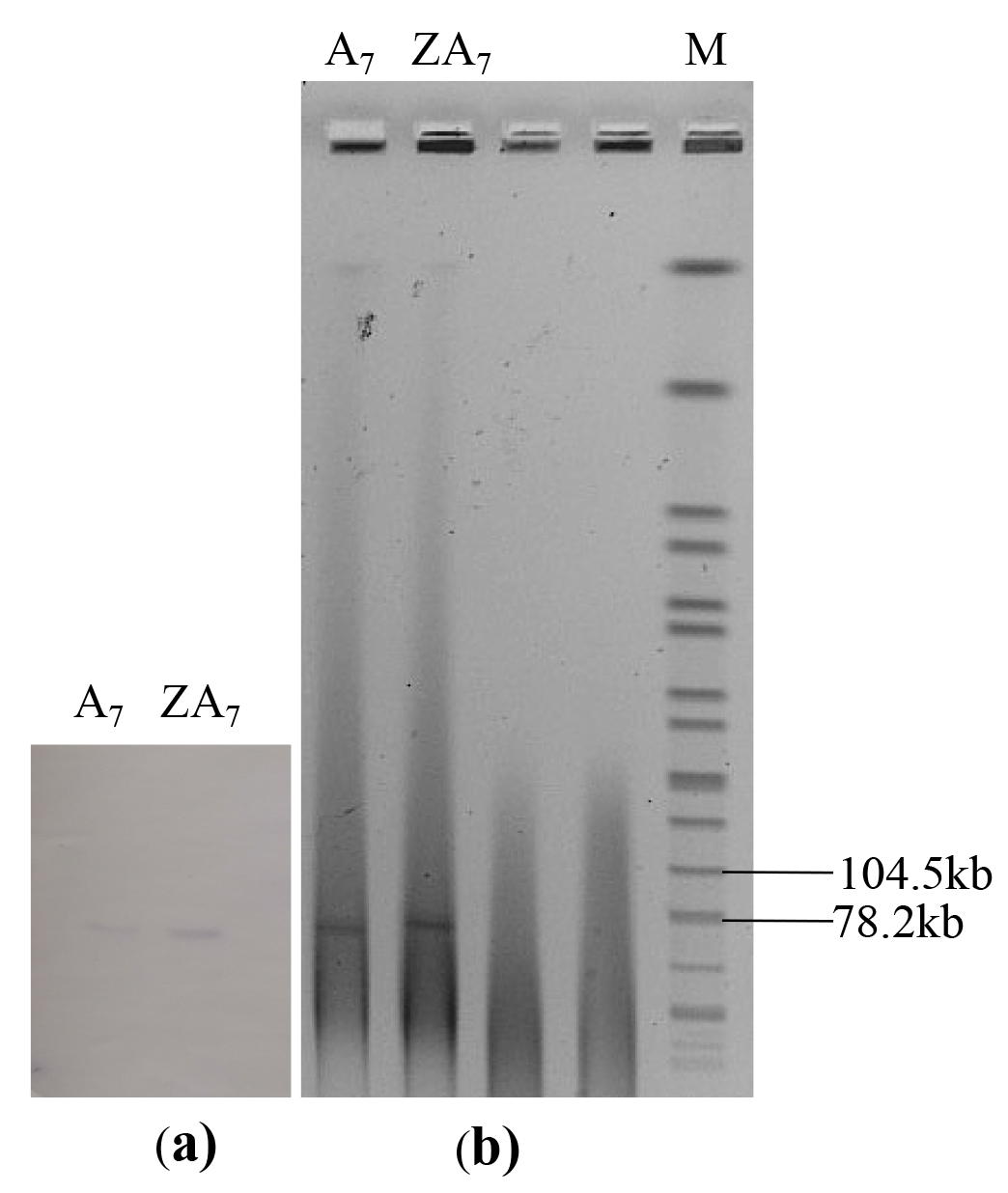

Supplement: Supplementary Figure S1 — (a) S1-PFGE of E. coli A2 and its transconjugant ZA2. (b) Southern hybridization of E. coli A2 and ZA2 with the tet(M) gene as the probe. Marker, Salmonella Braenderup H9812. [file Data_Sheet_1.ZIP › Supplementary files/FIGURE.S2.tif]
